# Lycorine Alkaloid and *Crinum americanum* L. (Amaryllidaceae) Extracts Display Antifungal Activity on Clinically Relevant *Candida* Species

**DOI:** 10.3390/molecules27092976

**Published:** 2022-05-06

**Authors:** Lorene Coelho Silva, Amabel Fernandes Correia, João Victor Dutra Gomes, Wanderson Romão, Larissa Campos Motta, Christopher William Fagg, Pérola Oliveira Magalhães, Dâmaris Silveira, Yris Maria Fonseca-Bazzo

**Affiliations:** 1 Department of Pharmacy, Health Sciences School, University of Brasília (UnB), Campus Darcy Ribeiro, Asa Norte, Brasilia 70910-900, Brazil; lorenecoelho@hotmail.com (L.C.S.); dutra.joaovictor@gmail.com (J.V.D.G.); perolamagalhaes@unb.br (P.O.M.); damaris@unb.br (D.S.); 2 Central Laboratory of the Federal District (LACEN-DF), Lotes O e P, Sgan 601, Asa Norte, Brasília 70830-010, Brazil; amabelfernandes@yahoo.com.br; 3 Federal Institute of Espíırito Santo, Vila Velha 29106-010, Brazil; wandersonromao@gmail.com; 4 Petroleomic and Forensic Laboratory, Department of Chemistry, Federal University of Espírito Santo, Vitória 29075-910, Brazil; larissacmotta@gmail.com; 5 Department of Botany, Institute of Biological Science, School of Pharmacy, Faculty of Ceilândia, University of Brasília, Brasilia 70910-900, Brazil; acaciafagg@gmail.com

**Keywords:** Amaryllidaceae, antifungal activity, *Candida* spp., alkaloid

## Abstract

*Candida* species are the main fungal agents causing infectious conditions in hospital patients. The development of new drugs with antifungal potential, increased efficacy, and reduced toxicity is essential to face the challenge of fungal resistance to standard treatments. The aim of this study is to evaluate the in vitro antifungal effects of two crude extracts of *Crinum americanum* L., a rich alkaloid fraction and lycorine alkaloid, on the *Candida* species. As such, we used a disk diffusion susceptibility test, determined the minimum inhibitory concentration (MIC), and characterized the components of the extracts using Electrospray Ionization Fourier Transform Ion Cyclotron Resonance Mass Spectrometry (ESI FT-ICR MS). The extracts were found to have antifungal activity against various *Candida* species. The chemical characterization of the extracts indicated the presence of alkaloids such as lycorine and crinine. The Amaryllidaceae family has a promising antifungal potential. Furthermore, it was found that the alkaloid lycorine directly contributes to the effects that were observed for the extracts and fraction of *C. americanum*.

## 1. Introduction

Fungi of the *Candida* genus are constituents of the human microbiota. They are commonly present in the skin, mucous membranes, and in the oral, gastrointestinal, and genitourinary cavities as commensal organisms [1]. *Candida* species are the main fungal agents causing infectious conditions in patients in the hospital environment, and candidiasis is a clinical challenge for the survival of patients with serious diseases and those in the postoperative period [2].

Clinically, candidiasis is an opportunistic infection manifesting superficially or in a systemic way [3,4]. The main *Candida* species associated with systemic candidiasis are *Candida* *albicans*, *Candida* *glabrata*, *Candida* *parapsilosis*, *Candida* *tropicalis*, and *Candida* *krusei*. The most frequent etiological agent, however, is still *C. albicans*. Over the years, *C. albicans* frequency has decreased; they accounted for approximately 57.4% of cases during 1997–2001, dropping to 46.4% in 2015–2016, and after that, non-albicans *Candida* spp. have been shown to be greater than 50% [5]. In addition, new *Candida* species have been reported. Since 2009, *Candida* *auris* has been identified worldwide and is associated with infections and outbreaks in healthy environments [6]. In the USA, *C. auris* has been reported since 2015, and its prevalence is increasing to date. According to the Centers for Disease Control and Prevention (CDC), the number of cases in 2018 increased by 318% compared to the average number of cases notified from 2015 to 2017. CDC data also indicate that at least 90% of *C. auris* isolates are resistant to at least one of the classic antifungals, and approximately 30% are resistant to at least two antifungals [7].

To treat *Candida* infections, drugs that can be grouped into four classes are used according to their mechanisms of action. Azoles, polyenes, and echinocandins are antifungal agents that act on the cell membrane, either by interfering with ergosterol biosynthesis, by forming channels that favor ionic imbalance, or by inhibiting the synthesis of β-(1,3)d-glucan (a cell wall polysaccharide) [8,9,10]. On the other hand, fluoropyrimidines act by harming nucleic acid biosynthesis through the formation of toxic antimetabolites, leading to an interruption in protein synthesis [9,10].

The emergence of drug-resistant *Candida* species is a growing health concern worldwide. For example, *C. krusei* has intrinsic resistance to fluconazole, with an overall resistance rate of 78.3%, but primary resistance to this antifungal is rare for *C. albicans* (1.4%), *C. parapsilosis* (3.6%), and *C. tropicalis* (4.1%). In terms of resistance to echinocandins, *C. parapsilosis* has a unique intrinsic resistance to these drugs, with higher minimum inhibitory concentration (MIC) values than other common *Candida* species [8,11]. *C. auris*, however, is the only species with isolates resistant to all four classes of human antifungals [8].

Although significant progress has been made towards identifying target structures for drugs in fungi, the search for new sources of treatment for fungal infections is urgently needed. Medicinal plants have been recognized for their promising potential [12]. The development of new drugs and molecules with antifungal potential, with increased efficacy and decreased toxicity, is essential for addressing the challenge of fungal resistance to standard treatments [9,13]. Studies have demonstrated the effectiveness of plant metabolites as potent antifungal agents. These substances have been shown to inhibit growth and alter the virulence of different *Candida* species, both in planktonic and hyphal forms, and during biofilm formation [10,14,15,16,17].

Lycorine, a natural alkaloid from the Amaryllidaceae family, has shown promising antifungal effects [18]. Yang et al. (2019) explored the antifungal activity of lycorine hydrochloride against *C. albicans*, finding the (MIC) of lycorine to be 64 μM. Below its MIC, lycorine demonstrated the ability to inhibit virulence factors by suppressing adhesion, biofilm formation, morphological transition, and the production of extracellular phospholipase and exopolymeric substances [19].

*Crinum* species extracts have been used in folk medicine to treat fever, pain, swelling, wounds, cancer, and malaria [20]. The biological activities of the *Crinum* species, including antitumor, immunostimulant, analgesic, antiviral, antibacterial, and antifungal activities, are mainly attributed to their alkaloids, which can be isolated from the bulbs, leaves, roots, and flowers [20,21]. These alkaloids have cytotoxic and antiviral activities, and a wide range of other physiological effects, such as antibacterial, anti-inflammatory, and acetylcholinesterase inhibition [22]. Recently, our research group investigated the activity of *Crinum americanum* L. (Amaryllidaceae) extracts and fractions on acetylcholinesterase enzyme inhibition, as well as their chemical composition by gas chromatography-mass spectrometry analysis. The chromatographic results combined with NMR experiments led to the identification of lycorine as a biomarker for this species [23]. However, to date, no data have described the antifungal effects of *C. americanum* extract on *Candida* species.

Therefore, this study evaluates the in vitro antifungal effects of two different crude extracts of *C. americanum*, an alkaloid-rich fraction and lycorine alkaloid, on *C. albicans*, *C. auris*, *C. krusei*, and *C. parapsilosis*. In addition, we chemically characterize the extracts and alkaloid-rich fraction by electrospray ionization Fourier transform ion cyclotron resonance mass spectrometry (ESI FT-ICR MS).

## 2. Results

### 2.1. Disk Diffusion Susceptibility Test

To determine the antifungal activity of the selected extracts and fractions in a qualitative manner, the National Committee recommends a disk diffusion susceptibility test for clinical laboratory standards (NCCLS) [24]. Thus, this test was used to screen the selected extracts and fractions.

*Crinum americanum* extracts showed antifungal activity against *Candida* spp. Particularly, the ethyl acetate fraction from bulbs (B_EAF) showed statistically significant results, with a higher inhibition halo than the control antibiotic, amphotericin B, for all strains tested. Additionally, the ethanolic extract from leaves (L_EE) and the ethanolic extract from bulbs (B_EE) also presented similar results for *C. albicans* and *C. auris*, in that they were superior to the results obtained for the control.

**Figure 1:**
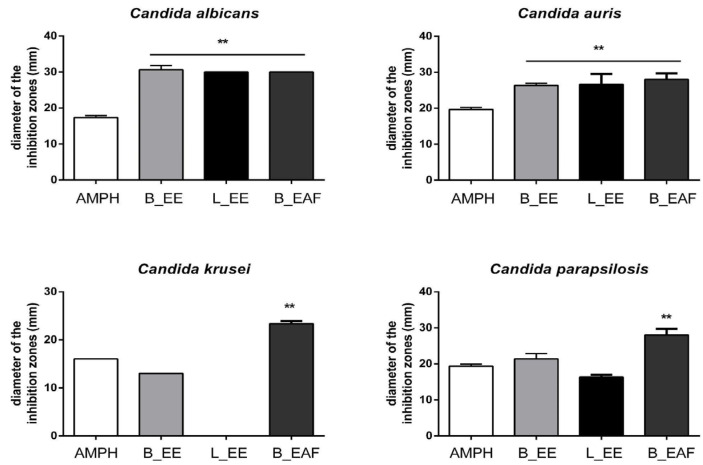
Disk diffusion susceptibility profile of *Candida* spp. to plant extracts. The diameter of inhibition halo (mm) values were expressed as the mean and standard deviation (n = 3). The values were compared with the amphotericin B group (AMPH, positive control) by an analysis of variance (ANOVA) with Dunnett’s post-test. ** Significant differences were considered as *p*-values of less than 0.05.

The measurements depicted in Figure 1 correspond to the extracts’ inhibition areas.

### 2.2. Minimum Inhibitory Concentration (MIC)

The minimum inhibitory concentration (MIC) assay was used to quantitatively determine the susceptibilities of microorganisms, that is, this test determined the lowest concentration of each extract to inhibit fungal growth. In this study, we performed MIC assays using lycorine to evaluate its antifungal potential. Table 1 summarizes the MIC values of the extracts and lycorine against *Candida* spp., as well as the results obtained with the positive and negative controls.

The results demonstrate that B_EAF had the lowest concentration needed for activity against all the *Candida* strains tested; B_EAF had better performance against both *C. albicans* and *C. krusei*, with a significant MIC of approximately 86 µg/mL. B_EAF had an MIC of 172 µg/mL for *C. parapsilosis*, and *C. auris* was the least susceptible to B_EAF, with an MIC of 344 µg/mL.

Additionally, lycorine demonstrated antifungal potential against the *Candida* species tested. The MIC of lycorine was lower than that of the tested extracts.

### 2.3. ESI (+) FT-ICR MS Analysis

**Figure 2:**
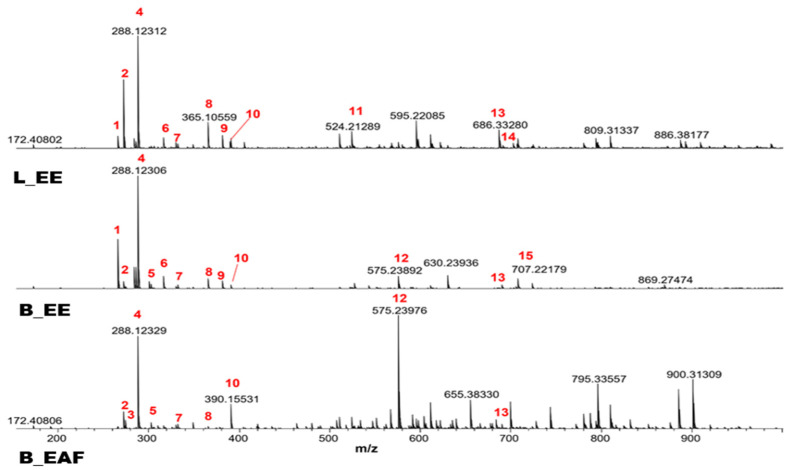
ESI (+) FT-ICR MS spectra of L_EE, B_EE, and B_EAF extracts of *Crinum americanum* L. (Amaryllidaceae). The identification of compounds according to the numbering in this figure is outlined in Table 2.

Figure 2 demonstrates the chromatograms of the extracts obtained from ESI (+) FT-ICR MS analysis. Table 2 describes the nominal identification of the main compounds, theoretical and measured values of m/z, molecular formula, mass error (ppm), and DBE (double bond equivalent).

The results of ESI (+) FT-ICR MS analysis indicates the presence of alkaloids, such as lycorine and crinine, already described in the literature. Moreover, we identified in all extracts naphtomycin E, but naphtomycin D was identified only in L_EE.

In L_EE, the presence of lycobetaine/ungeremine, crinine, lycorine or flexinine, homolycorine, 1-O-acetyllycorine, and cripowellin B was observed. In B_EE, lycobetaine/ungeremine, crinine, lycorine, flexinine, crinamine, 8-O-demethyl-homolycorine, homolycorine, 1-O-acetyllycorine, and lycorine dimers were detected. Similarlly, in B_EAF, crinine, norgalanthamine, lycorine or flexinine, crinamine or 8-O-demethyl-homolycorine, 1-O-acetyllycorine, and lycorine dimers were found. The alkaloid norgalanthamine was only found in B_EAF and cripowellin B was only present in L_EE.

## 3. Discussion

The ESI (+) FT-ICR MS results indicate the presence of alkaloids, as described in the literature. A recent study published by the same research group described the chemical profile of these extracts of *C. americanum* determined by CG/MS and RMN techniques [23].

In all samples, the lycorine, crinine, and 1-O-acetyl-lycorine were simultaneously identified. The compound with an m/z of 288.12303, corresponding to the [C_16_H_17_NO_4_ + H]+ ion, was identified as lycorine and that with an m/z of 272.12812 as crinine [C_16_H_17_NO_3_ + H]+. Both are frequently reported in several of the Amaryllidaceae family species, especially in the *Crinum* species, such as *Crinum x amabile* Donn ex Ker Gawl., *Crinum delagoense* I. Verd., *Crinum lugardiae* NEBr., *Crinum macowanii* Baker, and *Crinum moorei* Hook f. [21,22,25]. In the literature, 1-O-acetyl-lycorine has been identified as a potent acetylcholinesterase inhibitor [26]. However, no data on its antifungal potential have been described to date. Another substance was identified in the three extracts, with an m/z of 390.15501 and a molecular formula of [C_20_H_23_NO_7_ + H]+. However, no chemical structures have been proposed to date.

In extracts from leaves (L_EE) and bulbs (B_EE), we also identified homolycorine, with an m/z of 316.15433 and [C_18_H_21_NO_4_ + H]+ molecular formula, and licobetaine (m/z 266.08117) with the molecular formula [C_16_H_1_1NO_3_ + H]+. Homolycorine-type alkaloids are present in several genera of the Amaryllidaceae family, and the structural characteristics of these compounds make them strong candidates for the development of anticancer drugs, in addition to having moderate acetylcholinesterase inhibitory activity [27,28]. Evidente et al. (2004) tested hippeastrin, a representative of the homolycorine group, against *C. albicans* and obtained moderate antifungal activity, with an MIC of 125 μg/mL. Licobetaine is also an Amaryllidaceae alkaloid [29]. Barthelmes et al. (2001) reported that this alkaloid has in vitro inhibitory properties and significant cytotoxic activity against several types of carcinomas in mice [30].

Another alkaloid identified in both the extract of bulbs (B_EE) and ethyl acetate fraction from bulbs (B_EAF) was crinamine (m/z 302.13868), with the molecular formula [C_17_H_19_NO_4_ + H]+. Crinamine has already been identified in *Crinum jagus* (J.Thomps) Dandy bulbs and showed strong activity against *Bacillus subtilis* and *Staphylococcus aureus*. However, there was no confirmation of the antifungal activity of the alkaloid, which was tested against *Trichophyton mentagyrophytes* and *Aspergillus flavus* [31].

Norgalantamine (*m/z* 274.14377) was identified in the ethyl acetate fraction of the bulbs (B_EAF), using the molecular formula [C_16_H_19_NO_3_ + H]+. This alkaloid has demonstrated inhibitory activity against both cholinesterases (AChE and BChE) and may be a promising anti-Alzheimer molecule for future experiments [32].

In extracts from leaves (L_EE), cripowellin B (m/z 524.21289) was identified as having the molecular formula [C_25_H_33_NO_11_ + H]+. This alkaloid has already been described by Velten et al. (1998) [33] when it was isolated from the bulb of the species *Crinum* x *powellii* Baker. In a previous study, cripowellin B was also isolated from *C. americanum* and showed potent anti-plasmodial activity, in addition to its antiproliferative activity against human cancer cell lines [34,35]. A study carried out with the leaves of *C. americanum* identified known alkaloids, highlighting crinamine as a significant proportion of its components [36].

**Figure 3:**
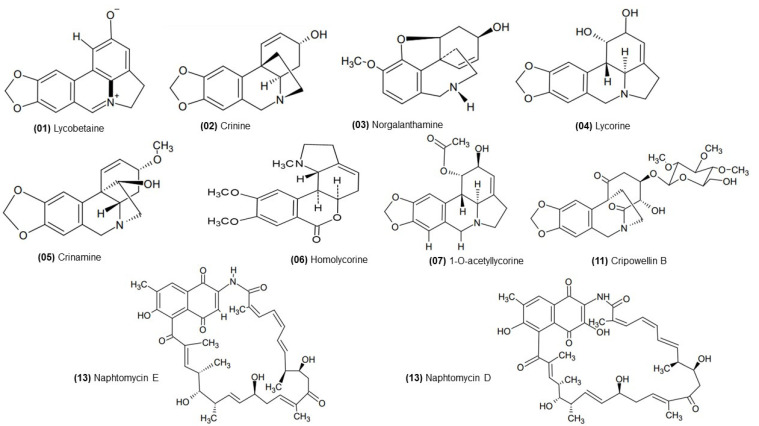
Compounds identified in the bulb and leaf extracts of *Crinum americanum* L.

In addition to the alkaloids, we identified in our extracts naphtomycin D (m/z 702.32727, [C_40_H_47_NO_10_ + H]+) and naphtomycin E (m/z 686.33236 and [C_40_H_47_NO_9_ + H]+). These compounds are products of the metabolism from endophytic organisms, as previously mentioned in the literature, for plants of the Amaryllidaceae family [37]. Lu and Shen (2007) reported the presence of similar substances in endophytic fungi [38]. Naphthomycins A, E, and K were isolated from commensal *Streptomyces* spp. Structural elucidation was performed by analyzing NMR and MS data [38]. Figure 3 shown the chemical structures of the identified compounds.

Determining the antifungal activity of constituents of the Amaryllidaceae family was the objective of a review by Nair and Staden (2018) [39]. In their review, the authors described findings related to trials conducted with nearly 40 constituents of the family, mainly isoquinoline alkaloids, which were tested against approximately 50 fungal pathogens. Promising results, with units as small as µg/mL, have been previously reported. Seven alkaloid groups were representatives of the class of compounds (phenanthridone, lycorane, crinane, galanthamine, tazettine, montanine, and homolycorine) [39]. In this context, we used *Candida* strains to evaluate the antifungal activity of *C. americanum* extract. The performance of the ethyl acetate fraction from bulbs (B_EAF) showed statistically significant results, with a higher inhibition halo than the control antibiotic, amphotericin B, for all strains tested. The extracts L_EE and B_EE also presented positive results for activity against *C. albicans* and *C. auris* and were superior to the results obtained for the control.

Alawode et al. (2021) performed tests with the methanolic extract of *C. jagus*, using the agar diffusion method, and demonstrated antifungal activity against *C. albicans*, *Aspergillus niger*, and *Penicillium notatum* with an inhibition zone of 20 mm at 200 mg/mL [40]. Another study with *C. jagus* showed that the methanolic extract obtained from this species exhibited an antimicrobial effect in vitro at a concentration of 100 mg/mL; the extract had the largest diameter of inhibitory zone against *Bacillus subtilis* (25 mm), *Staphylococcus aureus* (21 mm), and *C. albicans* (14 mm) [41]. The concentration used by Udegbunam et al. (2015) was similar to that which was used in our tests (100 mg/mL) [41]. In our results, the inhibition halos were even more significant for *C. albicans*, reaching > 30 mm. Therefore, our extracts demonstrated more significant antimicrobial activity than that reported in the literature for other *Crinum* species.

To the best of our knowledge, there are no published data on the activity of extracts from species of the Amaryllidaceae family against *C. auris*, suggesting that our results are innovative in this regard. However, other natural products have also been shown to be active against *C. auris*. A study on α-cyperone, which is the main component in the rhizome of the plant *Cyperus rotundus* L., showed that it prevented the growth of *C. auris* at concentrations of 150 µg/mL and 300 µg/mL [42]. Promising results were obtained in tests with 6-shogaol, a dehydrated product of 6-gingerol, extracted from *Zingiber officinale*; the compound exhibited antifungal and anti-biofilm activity by inhibiting biofilm formation and eradicating *C. auris* biofilms [16]. Even so, lycorine, a representative Amaryllidaceae alkaloid, achieved a higher activity than α-cyperone and 6-gingerol, with an MIC of 40.6 µg/mL.

The largest group of Amaryllidaceae alkaloids, the lycorine skeleton alkaloids, are known for a wide range of biological properties, with lycorine being the most studied alkaloid in fungal pathogenesis assays [39]. In the present study, lycorine was found to be active against all the fungal strains tested. The most susceptible strain to the pure compound was *C. parapsilosis* with an MIC of 20.3 µg/mL, followed by *C. albicans*, *C. krusei*, and *C. auris*. Lycorine and vittatine alkaloids were also tested for their ability to inhibit yeast growth, and lycorine proved to be very active, with final IC_50_ values ranging from 0.89 to 28.5 μg/mL [43]. In the present study, the MIC of lycorine against *C. albicans* (40.6 μg/mL) was similar to that reported by Ločárek et al. (2015) [27]; the authors obtained an MIC of 64 μg/mL for lycorine against a clinical isolate of *C. albicans*. Morphological studies performed by Toenjes et al. (2009) evaluated the ability of lycorine to inhibit the transition from the blastospore to hyphal state in *C. albicans* cultures [17]. The virulence of *C. albicans* largely depends on its ability to interconvert between various morphological states, including blastospores, pseudohyphae, and hyphae, which in turn are regulated by various cellular and environmental factors.

In the assay involving 480 molecules from the ICCB collection at Harvard University, lycorine was one of fifty-three molecules exhibiting cytotoxic effects on a clinical isolate of *C. albicans*, tested at 37 °C in a Spider medium at 130 μM over 4 h [17]. The authors emphasized that cytotoxic molecules, including lycorine, could play powerful roles in antifungal therapy and, therefore, could be excellent starting points for developing new antifungals. However, the effects of lycorine on *C. albicans* morphology were shown to be a consequence of cytotoxicity, rather than the inhibition of the transition from blastospores to hyphae [17].

Our assays did not evaluate interference at the structural level, and further tests are needed to assess the mechanism of action of lycorine against *Candida* species. Therefore, our MIC results for the extracts and lycorine are consistent with those described in the literature. An activity evaluation mechanism is needed to elucidate the lycorine action pathway in *Candida* strains. Furthermore, synergism tests with already established antifungal treatments may provide evidence of the antifungal properties of various compounds.

## 4. Materials and Methods

### 4.1. Plant Collection

*Crinum americanum* L. was collected from Brasília, DF, Brazil, and identified by a botanist (C.W.F.). A voucher specimen (Fagg 2474) was deposited at the University of Brasília (UnB) Herbarium (UB).

### 4.2. Preparation of Extracts and Fraction

The bulbs and leaves were dried in a forced-air circulation oven (Solab, model SL-102, Piracicaba, SP, Brazil) and cut into small pieces with scissors. The extracts were prepared as previously described by Gomes et al. (2022) [23]. Ethanolic extracts (L_EE and B_EE): The extracts were prepared by maceration for 72 h at a ratio of 1:10. First, hexane was used for extraction, and after three 72 h cycles of extraction, the residue was extracted using ethanol under the same extraction conditions. The extractive solutions were filtered and the solvents were removed by rotary evaporation at 40–45 °C. Ethyl acetate fractions (B_EAF): The residue from ethanol extraction was subjected to acidic extraction with 0.01 M hydrochloric acid. After boiling and cooling, the mixture was filtered and neutralized (pH 7–8) using 25% ammonium hydroxide (*v*/*v*). Subsequently, the neutralized aqueous solution was subjected to liquid–liquid extraction with ethyl acetate (3 × 60 mL). The ethyl acetate fractions (B_EAF) were obtained after removing the solvent from the ethyl acetate solution by rotary evaporation, under reduced pressure.

### 4.3. Antifungal Disk Diffusion Susceptibility Testing

The assay was performed using the disk diffusion method adapted from that described in document M44-A of the Clinical and Laboratory Standards Institute (CLSI) of the United States of America [24]. ATCC strains of *Candida albicans* (ATCC 90028), *Candida krusei* (ATCC 34135), and *Candida parapsilosis* (ATCC 22019) were used. For *Candida auris*, a control strain from the Department of Mycology of the Central Laboratory of the Federal District (LACEN–DF) was used and identified using matrix-assisted laser desorption ionization time-of-flight (MALDI-TOF) mass spectrometry. *Candida* spp. were maintained by successive replications in Sabouraud dextrose agar medium, undercooled (4 °C) and cryopreserved in Sabouraud dextrose broth with glycerol (20%) at −80 °C. For the tests, the strains were sub-cultured in Sabouraud medium and incubated for 24–48 h at 37 °C.

B_EE, L_EE, and B_AEF were solubilized at a concentration of 100 mg/mL in ethanol. Then, 10 µL of each sample was applied to the center of filter paper discs (6 mm diameter). The discs were kept in a Petri dish at room temperature for 24 h to dry and completely evaporate the solvent. Negative control discs with solvent only were prepared in the same manner as described for the extracts. Amphotericin B disks (100 mg/mL) were used as the positive controls.

Mueller–Hinton agar (HiMedia, Mumbai, India) was used for the disk diffusion method. The fungus was inoculated by applying the streak depletion technique with rotation of the plate at an angle of 60°, three times. Discs containing extracts were applied to the inoculated surface. The inverted plates were incubated at 35 °C (±2 °C) for up to 48 h [24]. The same procedure was performed in the control group. All extracts and controls were tested in triplicate. The mean diameter of inhibition zones was calculated [44] and the results were read using a caliper to measure the diameter of the inhibition zones.

### 4.4. Determination of Minimum Inhibitory Concentration (MIC)

MIC evaluation was performed using the microdilution plate method adapted from Mlozi et al. (2020) [45]. For the MIC assay, 96-well plates were used, and experiments were performed in triplicate. All samples were diluted in ethanol and tested in the following concentration ranges (intermediate values of range concentrations are described in the Supplementary Material Table S1):

Assays using *Candida albicans* and *Candida auris*: 51.50–0.03 mg/mL B_EE, 54.00–0.03 mg/mL L_EE, and 22.00–0.01 mg/mL B_EAF.

Assays using *Candida parapsilosis*: 56.50–0.03 mg/mL B_EE, 54.00–0.03 mg/mL L_EE, and 44.00–0.02 mg/mL B_EAF.

Assays using *Candida krusei*: 56.50–0.03 mg/mL B_EE and 44.00–0.02 mg/mL B_EAF.

Lycorine hydrochloride (LYC) (CAS: 2188-68-3; Sigma) was tested under the same conditions. LYC concentrations were between 0.32 and 650 µg/mL, as described by Ločárek et al. (2015) [27].

The fungal inoculum was prepared from subcultures of the strains on Sabouraud dextrose agar (HiMedia, Mumbai, India) at 35 °C (± 2 °C) for 24 to 48 h. Microorganisms were suspended and homogenized by vortexing in approximately 5.0 mL of 0.85% sterile saline solution, to obtain a suspension equivalent to 0.5 on the McFarland scale (1 × 10^6^ to 5 × 10^6^ cells/mL) [24]. The standardization of the colony suspension of each strain was confirmed using a turbidimeter.

Initially, 100 µL of brain heart infusion broth (BHI) was added to each well. each sample was diluted in BHI. Then, 100 µL of concentrated test sample was added to the first well of a 12-well sequence. After homogenization, serial dilutions of 1:2 were performed. Then, 100 µL of fungal inoculum was added to each well. All samples and controls were analyzed in triplicate. Finally, each plate was sealed with the appropriate adhesive and incubated at 37 °C for 24 h. After this period, 15 µL of freshly prepared resazurin solution (0.01%) was added to each well. Homogenization was performed using a shaking table for 20 min. Resazurin, a dye (fenoxazin-3-one) that indicates oxide reduction, has a blue color in its oxidized form. Its reduced form is pink in color [46]. The sealed plates were incubated at 37 °C for 18 to 24 h. The reading was performed by visualizing the change in color of the wells. In this study, the lowest concentration was that which maintained the initial color (blue), indicating the absence of microbial growth, or the first dilution in which the color changed from blue to subtly pink. The color change indicates a reduction in resazurin and, therefore, microbial growth.

### 4.5. ESI (+) FT-ICR MS Analysis

The ethanol extracts and ethyl acetate fraction of the bulb were analyzed by electrospray ionization Fourier transform ion cyclotron resonance mass spectrometry (ESI FT-ICR MS). As such, the extracts were solubilized in 1 mL of methanol (Vetec^®^ Química Fina Ltd., Brazil, > 99.8%). The flow rate was 5 μL/min. The analysis was performed using an electrospray ionization (ESI) source operated in positive mode, model 9.4 T Solarix, Bruker Daltonics (Bremen, Germany), in a mass range of m/z 150–1000 Da. The parameters of the ESI source (+) were: (i) capillary voltage (cone): 3500–4100 V; (ii) endplate displacement = 500 V; (iii) temperature and drying gas flow: 250 °C and 2 µL/min; (iv) nebulizer gas pressure: 1 bar; (v) skimmer: 15 V; and (vi) collision voltage: (±) 1 V. In ionic transmission, the hexapole ion accumulation time and the flight time were 0.02 s and 0.9 ms, respectively. Each spectrum was acquired by accumulating 16 time-domain signal scans at 4 M (megapoints). All mass spectra were externally calibrated using an arginine solution (m/z 150–1000). The resolving power was approximately 500,000 at an m/z of 400, providing precise molecular formula assignments for the individually charged molecular ions. FT-ICR mass spectra were acquired and processed using software for data analysis (Bruker Daltonics, Bremen, Germany), in order to determine the proposed structures for each molecular formula, which were assigned using the ChemSpider database (http://www.chemspider.com, accessed on 16 November 2021) and PubChem (https://pubchem.ncbi.nlm.nih.gov/, accessed on 16 November 2021).

### 4.6. Statistical Analysis

Microsoft Office Excel^®^ 2022 software and GraphPad Prism^®^ Version 6.0 were used for statistical analysis. The results are expressed as the average plus the standard deviation. An analysis of variance (ANOVA), followed by Dunn’s multiple comparison tests, were used for statistical analysis. Differences were considered significant when *p*-values were less than 0.05.

## 5. Conclusions

Plants in the Amaryllidaceae family have antifungal potential, and in the future, research will focus on their use in different formulations. However, it is necessary to briefly establish the mechanism of action for each extract, expand knowledge about the potential of each component, and define how they affect microorganisms. There are reports in the literature regarding the antifungal activity of plants of the Amaryllidaceae family, especially species of the genus *Crinum*. Even so, our study extended this investigation, and the results obtained can be a basis for further studies relying on bioprospecting of antifungal alkaloids. Furthermore, it was found that the alkaloid lycorine directly contributes to the effects observed for the extracts and fractions of *Crinum americanum* L.

## Figures and Tables

**Table 1:** MIC values of the extracts and lycorine against *Candida* spp. strains (results in µg/mL).

*Candida* Strain	CIM L_EE	CIM B_EE	CIM B_EAF	CIM Lycorine	Positive Control	Negative Control	AMPH Control
*C. auris*	805	422	344	162.5	+	-	-
*C. albicans*	805	422	86	40.6	+	-	-
*C. krusei*	221	NA	86	81.3	+	-	-
*C. parapsilosis*	441	422	172	20.3	+	-	-

Legend: (+) = growth; (-) = no growth; NA—not applicable.

**Table 2:** Identification of the alkaloids by ESI (+) FT-ICR MS in the extracts from leaves (L_EE), bulbs (B_EE), and ethyl acetate fraction of bulbs (B_EAF) from *Crinum americanum* L.

Num.	Chemical Compound	L_EE	B_EE	B_EAF	Molecular Formula	Theory m/z	Measured m/z	Error ppm	DBE
1	Lycobetaine/Ungeremine	X	X	-	[C_16_H_11_NO_3_ + H]+	266.08117	266.08110	0.26	12
2	Crinine	X	X	X	[C_16_H_17_NO_3_ + H]+	272.12812	272.12808	−0.33	9
3	Norgalanthamine	-	-	X	[C_16_H_19_NO_3_ + H]+	274.14377	274.14378	−0.02	8
4	Lycorine or Flexinine	X	X	X	[C_16_H_17_NO_4_ + H]+	288.12303	288.12312	−0.31	9
5	Crinamine or 8-O-demethyl-homolycorine	-	X	X	[C_17_H_19_NO_4_ + H]+	302.13868	302.13872	−0.11	9
6	Homolycorine	X	X	-	[C_18_H_21_NO_4_ + H]+	316.15433	316.15414	−0.02	9
7	1-O-acetyllycorine	X	X	X	[C_18_H_19_NO_5_ + H]+	330.13360	330.13379	−0.47	10
8	Sucrose or isomers (sodium adduct)	X	X	X	[C_12_H_22_O_11_ + Na]+	365.10543	365.10545	−0.04	2
9	Sucrose or isomers (potassium adduct)	X	X	-	[C_12_H_22_O_11_ + K]+	381.07937	381.07951	−0,47	2
10	-	X	X	X	[C_20_H_23_NO_7_ + H]+	390.15473	390.15501	−0.56	10
11	Cripowellin B	X	-	-	[C_25_H_33_NO_11_ + H]+	524.21264	524.21289	−0.48	10
12	Lycorine Dimer	-	X	X	2 [C_16_H_17_NO_4_ + H]+	575.23879	575.23933	−0.93	17
13	Naphtomycin E	X	X	X	[C_40_H_47_NO_9_ + H]+	686.33236	686.33242	−0.64	18
14	Naphtomycin D	X	-	-	[C_40_H_47_NO_10_ + H]+	702.32727	702.32807	−1.13	18
15	Sucrose Dimer or isomers (potassium adduct)	-	X	-	2 [C_12_H_22_O_11_ + Na]+	707.22164	707.22179	−0.21	2

## Data Availability

Not applicable.

## Supplementary Materials

The following supporting information can be downloaded at: https://www.mdpi.com/article/10.3390/molecules27092976/s1, Table S1: Sample concentrations on MIC plates.

## Sample Availability

Samples of the compounds are available from the authors.
