# Supplementary material for: Lycorine Alkaloid and Crinum americanum L. (Amaryllidaceae) Extracts Display Antifungal Activity on Clinically Relevant Candida Species
Source: Molecules. 2022 May 6;27(9):2976. doi: 10.3390/molecules27092976 (PMC9100883; doi:10.3390/molecules27092976)
Supplement: Supplementary file 1 [file molecules-27-02976-s001.zip › molecules-1681212-supplementary.pdf]

molecules

MDPI

Supplementary material

Table S1 - Sample concentrations on MIC plates.

	Candida albicans and Candida auris			*Candida parapsilosis*			*Candida Krusei*		All *Candida* species
**Position**	***B_EE**	***L_EE**	***B_EAF**	***B_EE**	***L_EE**	***B_EAF**	***B_EE**	***B_EAF**	****Lycorine**
**1**	51.50	54.00	22.00	56.50	54.00	44.00	56.50	44.00	650.00
**2**	25.75	27.00	11.00	28.25	27.00	22.00	28.25	22.00	325.00
**3**	12.88	13.50	5.50	14.13	13.50	11.00	14.13	11.00	162.50
**4**	6.44	6.75	2.75	7.06	6.75	5.50	7.06	5.50	81.25
**5**	3.22	3.38	1.38	3.53	3.38	2.75	3.53	2.75	40.63
**6**	1.61	1.69	0.69	1.77	1.69	1.38	1.77	1.38	20.31
**7**	0.80	0.84	0.34	0.88	0.84	0.69	0.88	0.69	10.16
**8**	0.40	0.42	0.17	0.44	0.42	0.34	0.44	0.34	5.08
**9**	0.20	0.21	0.09	0.22	0.21	0.17	0.22	0.17	2.54
**10**	0.10	0.11	0.04	0.11	0.11	0.09	0.11	0.09	1.27
**11**	0.05	0.05	0.02	0.06	0.05	0.04	0.06	0.04	0.63
**12**	0.03	0.03	0.01	0.03	0.03	0.02	0.03	0.02	0.32

*Unit: mg/mL

**Unit: µg/mL
